# Developmental plasticity: Bridging research in evolution and human health

**DOI:** 10.1093/emph/eox019

**Published:** 2018-02-05

**Authors:** Amanda J Lea, Jenny Tung, Elizabeth A Archie, Susan C Alberts

**Affiliations:** 1 Department of Biology, Duke University, Durham, NC 27708, USA; 2 Institute of Primate Research, National Museums of Kenya, Karen, Nairobi, Kenya; 3 Duke University Population Research Institute, Duke University, Durham, NC 27708, USA; 4 Department of Evolutionary Anthropology, Duke University, Durham, NC 27708, USA; 5 Department of Biological Sciences, University of Notre Dame, Notre Dame, IN 46556, USA

**Keywords:** developmental plasticity, early life effects, gene-environment interaction, epigenetics, evolution of plasticity, developmental origins of health and disease

## Abstract

Early life experiences can have profound and persistent effects on traits expressed throughout the life course, with consequences for later life behavior, disease risk, and mortality rates. The shaping of later life traits by early life environments, known as ‘developmental plasticity’, has been well-documented in humans and non-human animals, and has consequently captured the attention of both evolutionary biologists and researchers studying human health. Importantly, the parallel significance of developmental plasticity across multiple fields presents a timely opportunity to build a comprehensive understanding of this phenomenon. We aim to facilitate this goal by highlighting key outstanding questions shared by both evolutionary and health researchers, and by identifying theory and empirical work from both research traditions that is designed to address these questions. Specifically, we focus on: (i) evolutionary explanations for developmental plasticity, (ii) the genetics of developmental plasticity and (iii) the molecular mechanisms that mediate developmental plasticity. In each section, we emphasize the conceptual gains in human health and evolutionary biology that would follow from filling current knowledge gaps using interdisciplinary approaches. We encourage researchers interested in developmental plasticity to evaluate their own work in light of research from diverse fields, with the ultimate goal of establishing a cross-disciplinary understanding of developmental plasticity.

## INTRODUCTION

Early life environments can profoundly shape traits related to both human health and Darwinian fitness. For example, humans exposed to famine *in utero* exhibit higher rates of obesity, heart disease and schizophrenia in adulthood than siblings conceived under better conditions [1, 2]. In addition, children exposed to many adverse psychological events experience more physiological wear-and-tear by midlife, and live shorter lives on average than individuals exposed to few adverse events [3, 4]. Similar effects of early life conditions are observed in non-human vertebrates. In red deer and Asian elephants, individuals born during ecologically challenging periods experience faster reproductive senescence than individuals born during better times [5, 6], and in zebra finches and great tits, the availability of resources in early life predicts clutch size in adulthood [7]. Together, these studies provide just a few striking examples of the impact early life environments can have on later life traits.

The capacity of genetically similar individuals to produce substantially different phenotypes depending upon environmental conditions during early life (defined here as the period between conception and reproductive maturation, following [8]) is known as ‘developmental plasticity’. Because the impact of early conditions can be so dramatic, with potent effects on reproduction and survival, developmental plasticity is of central interest to multiple disciplines. Researchers in medicine, public health, psychology, economics, and sociology seek to understand the link between early conditions and adult health because of its relevance to disease treatment and prevention (reviewed in [9–14]). Concurrently, evolutionary biologists seek to understand the impact of early environments on traits related to Darwinian fitness, because this knowledge is important for understanding the evolution of complex traits and the selection pressures that shape them (reviewed in references [15–19]).

The parallel importance of early environmental effects in both health and evolutionary research presents an opportunity to forge a more complete, interdisciplinary understanding of developmental plasticity. We argue that realizing this opportunity should be a priority, but doing so is challenging because conflicting theories and terminology have arisen in the different fields in the absence of the cross-talk necessary to resolve them. Our goal here is thus to initiate and encourage increased connectivity, specifically by outlining key questions shared by health and evolutionary researchers, as well as approaches from diverse fields that could be used to address them. In doing so, we do not comprehensively review developmental plasticity research (we refer interested readers to many excellent, recent reviews [9–19]). Instead, we aim to spark excitement about work in three distinct areas where the potential for cross-disciplinary gain is high. In the first section, we discuss evolutionary explanations for developmental plasticity and highlight critical tests that distinguish between potential explanations. In the second and third sections, we shift our emphasis to focus on the molecular basis of developmental plasticity, including the contribution of genetic variation (see ‘Genetics and genomics of developmental plasticity’ Section) and the molecular mechanisms that mediate plasticity within an individual’s lifetime (see ‘The molecular mechanisms that mediate developmental plasticity’ Section). In each area, we discuss current knowledge gaps, promising approaches for filling these gaps and the gains in human health and evolutionary biology that would follow. Because of our focus on human health, we primarily draw on studies of humans and other mammals, but where relevant, we include important work from birds, insects, and other taxa. Finally, we provide a roadmap to terminology commonly used by health researchers and evolutionary biologists (Table 1), as well as a brief guide to evolutionary frameworks that are discussed in both communities (Box 1).

**Table 1.** Key terms in developmental plasticity research

Term	Range of definitions	Proposed usage
Selection	Abbreviation for ‘natural selection’ or ‘evolution by natural selection’. The process by which individuals with certain traits experience greater reproduction and/or survival than individuals without the trait (or with some alternate trait value). Under natural selection, genetic variants that contribute to superior phenotypes increase in frequency. Abbreviation for ‘selection bias’ and related phenomena such as ‘social selection’ and ‘health selection’. It occurs when some individuals or groups are more likely than others to be sampled, or when some individuals or groups are more likely than others to experience a given set of conditions. When these types of selection occur, the effects of early life conditions are observed in a non-random sample of individuals.	Avoid the abbreviation; specify whether the topic is natural selection, social selection, health selection, selection bias, and so on.
Heritable	Used to describe a trait for which a measurable proportion of total phenotypic variance is explained by genetic differences among individuals. A central concept in evolutionary biology, distinct from non-technical terms such as ‘inherited’. Sometimes used in the non-technical sense to mean that phenotypes of offspring and parents are correlated, without demonstration that this phenotypic similarity is due to genetic similarity.	Use the term ‘inherited’ instead of ‘heritable’ for meaning number 2, i.e., in the absence of data to support heritability in the technical sense.
Adaptation	Used to describe a trait that increases the average fitness of individuals that express it, relative to individuals that do not express the trait. Adaptive traits in this sense arise and persist through natural selection, and adaptation occurs over generations rather than within an individual’s lifetime. Used to describe short-term physiological adjustment to a current environment (e.g. the ability of the eye to adjust to different light levels). Adaptation in this sense occurs within the organism’s lifetime. Used to describe a trait that appears to be beneficial (i.e. a trait that appears to promote health or well-being in the environment of interest) even in the absence of direct evidence for fitness differences or heritability.	When using these terms, clarify whether evolutionary adaptation or short-term physiological adaptation is meant. If referring to evolutionary adaptation, the term should be reserved for traits with demonstrated (rather than assumed) fitness advantages in carriers, relative to other individuals in a population or species.
Maladaptation	Used to describe a trait that decreases the net average fitness of individuals that express it, relative to individuals that do not express the trait. Traits are not maladaptations simply because they impose costs; immediate costs of a trait may be offset by benefits that trait-bearers accrue at other stages of the life history, or may result from tradeoffs that allow survival at the cost of suboptimal phenotypes. True maladaptations (i.e. traits that impose net costs on the individuals that bear them) occur most often if the environment changes and a formerly neutral or adaptive trait becomes a liability. Used to describe a trait that appears to be detrimental to health or well-being in a particular environment. When this use is employed, net costs and benefits over the course of the lifetime, and potential tradeoffs between traits, are usually not considered.	Confine the use of this term to definition 1. In the absence of evidence that a trait is truly maladaptive, replace the term with another word, such as detrimental, and clarify that only immediate or short term costs are being considered.

Box 1. A brief guide to evolutionary explanations for developmental plasticity

‘Development constraints’ models posit that, in the face of early adversity, natural selection favors developmental strategies that promote immediate survival, even if other aspects of development are impaired. Thus, in the face of constraints, organisms ‘make the best of a bad job’ and follow developmental trajectories that avoid immediate death or impairment, but that may generate negative consequences later in life. For instance, the ‘silver spoon ‘effect’ occurs when exposure to favorable conditions during development produce fitness advantages in adulthood, and conversely when adverse conditions during development produce disadvantages later in life [21]. The ‘thrifty phenotype hypothesis’ posits that inadequate early nutrition triggers ‘nutritional thrift’, impairing the development of pancreatic function and pre-disposing the individual to metabolic disorders in adulthood [163]. This hypothesis was later updated to posit that metabolic disorders in later life would be milder if adult nutritional conditions closely matched those in childhood ([164], reviewed in [15]). However, evidence is limited for health gains following matched early life and adult nutritional environments (see ‘Tests of developmental constraints versus predictive response models’ Section ); thus we view the thrifty phenotype, at its core, as a hypothesis about constraints. The ‘allostatic load hypothesis’ invokes stressors—during development and throughout life—as challenges to which the organism must respond by modifying its physiology and behavior. The accumulation of such challenges results in increased susceptibility to disease [165]. This hypothesis is often overlooked in the developmental plasticity literature, but we include it here because, by explicitly invoking stressors during development as contributors to poor health in later life, it has the hallmarks of a development constraints model. Contrary to some interpretations, developmental constraints models are models of an adaptive process: a developmentally plastic organism will generally have higher fitness than one that is unable to alter any development processes in the face of environmental limitations [18].

 In contrast to constraints models, ‘predictive models’ posit that natural selection produces organisms that adjust their phenotype during development to optimize their performance in a future (as opposed to the current) environment. The standard ‘PAR’ model proposes that cues in early life trigger phenotypic responses that improve fitness at a later stage of development [9, 24, 25, 166]. This model, also called the ‘“external” PAR model’ (ePAR; [26, 34]), finds strong support in a few short-lived vertebrates and invertebrates [25, 39], weak support in a meta-analysis of 58 experimental plant and animal studies [39], and no or inconclusive support in the four species of long-lived vertebrates in which it has been tested [27–31]. Another problem for predictive models is that adaptive adjustments can only occur if a cue during development accurately predicts the adult environment [18, 33, 34], a condition that is rare for long-lived organisms. The recently proposed ‘“internal” PARr model’ (iPAR; [26]) offers a solution to this problem by suggesting that developmental responses to adversity are not adapted to future ‘external’ conditions, but rather to the predicted poor ‘internal’ somatic state of individuals growing up under adversity. The iPAR converges with developmental constraints models in invoking developmental tradeoffs as a primary driver of poor adult outcomes. However, it differs from constraints models by proposing that the developing organism will respond to these tradeoffs by maturing early to maximize reproduction under a shorter life expectancy ([26]; see [167, 168] for recent empirical tests of iPAR). The ‘adaptive calibration model’ (ACM), another recent, untested variant of predictive models, also predicts accelerated maturation when early adversity increases mortality risk [23, 32]. The ACM also invokes later life plasticity, making the difficult-to-test prediction that individuals may re-calibrate their physiological responses to adversity later in life in response to environmental conditions.

### The evolution of developmental plasticity

#### Shared interests, current knowledge, and gaps in knowledge

Dobzhansky’s famous message—that ‘nothing in biology makes sense except in the light of evolution’ [20]—has been taken to heart by researchers interested in developmental plasticity. The result has been a plethora of explanations for how natural selection has produced a trait, namely early environmental sensitivity, that can sometimes lead to detrimental health or fitness outcomes. These explanations fall into two broad categories, both of which assume a role for adaptive evolution (Box 1). However, the two categories of models differ in whether they view plastic responses as determined by immediate constraints or as anticipatory.

‘Developmental constraints models’ propose that, in resource-limited environments, developing organisms make tradeoffs to protect critical functions (e.g. investing in brain development at the expense of growth). These tradeoffs may improve the organism’s chance of survival in early life, but reduce long-term somatic quality and compromise adult health [8, 15, 21, 22]. In other words, natural selection may attempt to optimize overall fitness (relative to individuals that exhibit no plasticity) by accepting tradeoffs that carry long-term costs (relative to individuals who did not experience resource limitation). In contrast, ‘predictive models’ describe a situation in which environmental cues in early life predict the adult environment; hence organisms evolve the ability to adjust their phenotype during development to maximize fitness in the predicted adult environment ([9, 23–26]; see Box 1). Predictive models thus invoke the presence of ‘informational’ cues in early life that, over evolutionary history, have reliably predicted the nature of the adult environment [18]. If these early cues induce an incorrect predictive response (e.g. fail to correctly predict the adult environment), the resulting mismatch results in poor later life health.

Despite the conceptual impact of both developmental constraints and predictive models, few studies have attempted to empirically distinguish between them, especially in humans or other long-lived mammals (but see [27–31]). In addition, the last few years have brought new hypotheses that merge some aspects of constraints and predictive frameworks [23, 26, 32] (Box 1). Only recently have researchers attempted to identify the contexts in which predictive versus constraints-induced plasticity evolves [18, 33–38]. Expanding on these two areas of research—testing predictions from theory and identifying contexts that promote plasticity evolution—is critical for understanding the evolution and maintenance of early life effects, as well as the health costs incurred by early adversity. Below we discuss approaches for doing so, as well as the specific gains that would follow.

#### Tests of developmental constraints versus predictive response models

A critical test of predictive versus constraints models requires comparing the fitness of individuals born in high-quality environments with those born in low-quality environments, when both sets of individuals experience both high- and low-quality conditions as adults [15, 27, 29, 30, 39]. Under a predictive model, fitness will be maximized when individuals encounter matched early life and adult environments, whereas under a constraints model, individuals born in high-quality environments will consistently outperform individuals born in low-quality environments. Experiments that satisfy this ‘fully factorial design’ have now been conducted in dozens of short-lived insect, amphibian, reptile and plant species, with stronger support for constraints than predictive plasticity [39]. However, in a few striking cases, experimental manipulations of early life auditory cues that signal environmental quality have produced strong evidence for predictive plasticity (e.g. in red squirrels [40] and zebra finches [41]).

In humans, inferences about the evolution of developmental plasticity have been largely based on cross-population or between-cohort comparisons [42–45], as well as longitudinal studies that do not satisfy the fully factorial design. This limitation exists because identifying human populations that are appropriate for fully factorial, within-individual tests is challenging, as is obtaining individual-based, longitudinal data. Consequently, in spite of enthusiasm for the idea that mismatches between early and later life environments produce pathology in humans, evidence in support of this idea is very limited. Indeed, the only critical tests of predictive models in humans that we know of, in pre-industrial Finnish populations, find no support for predictive adaptive responses (PARs). Instead, these studies find that pre-industrial Finns born in poor environments exhibit fertility and survival detriments, rather than enhancements, when they re-encounter challenging environments in adulthood [20, 23].

Inspite of the challenges of studying developmental plasticity in long-lived species, several longitudinal studies of human populations exposed to varying levels of early adversity are underway [46–48]. With time, these studies have the potential to identify the environments and traits that are best explained by constraints versus predictive models. Meanwhile, long-term studies of long-lived mammals offer great potential for performing fully factorial tests, especially in cases where longitudinal data exist or are being collected. Already, three groups have leveraged data from well-studied populations (of roe deer, bighorn sheep and yellow baboons) to examine fitness outcomes when individuals naturally encounter environments that both match and mismatch their early life conditions [27, 30, 31]. All three studies found stronger support for developmental constraints than predictive plasticity, with no or limited support for predictive plasticity overall (see Box 1). Other long-term studies of wild mammals [49] are poised to contribute similar tests. In addition to circumventing limitations faced by human studies, such work will be important for interpreting the evolutionary history of developmental plasticity in a comparative framework, including the degree to which aspects of early life effects in humans are unique.

#### Identifying contexts in which predictive responses and developmental constraints evolve

Recently, several groups have used simulations and mathematical models to identify the contexts in which predictive plasticity should evolve [18, 33, 34, 50]. This work has highlighted two predictions relevant to human disease, as well as the evolution of plasticity more generally. First, when the environment varies stochastically on a timescale shorter than the organism’s generation time, early life conditions will be a poor predictor of the adult environment, and predictive plasticity will be unlikely to evolve [26, 33, 34, 51]. This situation is commonly encountered by long-lived animals, who often experience unpredictable heterogeneity in rainfall, food availability, and other environmental variables over the course of their multi-year lives [27–30]. Thus, we should expect predictive plasticity to be rare in these contexts. Second, organisms can only evolve plastic responses to environments they are repeatedly and consistently exposed to over evolutionary time, and responses to novel or atypical environments may generally be disadvantageous [52, 53] (Box 1) [33, 54]. If true, this prediction implies that for experimental studies focusing on extreme or novel early life challenges (e.g. exposure to recently introduced toxins), it may be inappropriate to interpret the results in an adaptive evolutionary framework.

Testing these two predictions—(i) that predictive responses are unlikely to evolve in stochastic environments and (ii) that highly novel early environments trigger maladaptive responses—is fundamental for assessing and refining current frameworks. Such tests have already begun in systems amenable to experimental evolution (e.g. fungal and bacterial species, as well as *Caenorhabditis elegans* and *Drosophila melanogaster*), and this work has supported the idea that different types of environmental predictability select for different types of plasticity [35, 38, 55–59]. For example, Dey et al. [35] exposed *C. elegans* to an environmental regime that predictably alternated between normoxia and anoxia every generation. Because the future environment could be reliably predicted from current conditions, populations evolved the ability to ‘prepare’ offspring for the alternate environment (by manipulating glycogen provisioning to their embryos).

Though compelling, evolutionary experiments of this type are infeasible for long-lived organisms, and alternative approaches are needed. Cross-taxon analysis can be used to test whether predictive plasticity evolves when future environments are predictable: once empirical tests have been performed in a sufficiently diverse set of species, researchers can examine the relationship between life history variation, ecological variation and predictive plasticity evolution. Amassing the required set of empirical tests is clearly a tall order, but points to an important goal for the research community. Testing the second theoretical prediction—that highly novel early environments trigger maladaptive responses (Table 1)—can be addressed with comparative data from species that encounter rapid environmental change or urbanization. For instance, studies that examine behavioral responses to human-induced environmental change are generating considerable data on the extent and nature of maladaptive responses to highly novel environments [60, 61]. This framework could be expanded to incorporate early life effects. Importantly, the prediction that novel environments trigger maladaptive responses would hold under both a constraints and predictive plasticity framework: both models assume plasticity has evolved through natural selection, a process that usually requires many generations of environmental exposure.

#### Gains from answering outstanding questions

For researchers interested in human health, understanding when predictive models do and do not explain developmental plasticity is essential for knowing what the optimal adult environment ‘looks like’ for each individual, and which mitigation strategies will best combat the effects of early adversity. Under a predictive model, health is maximized when early and adult environments are concordant, suggesting, for instance, that manipulating adult diet or lifestyle could mitigate the effects of undernourishment in early life. In contrast, if adult phenotypes arise from developmental constraints, ‘matching’ the adult environment to the early one will be unproductive or even detrimental, and intervention efforts should focus on improving early conditions. Sound theory and a robust body of empirical work are essential for understanding whether predictive plasticity is expected to be common or rare in long-lived species such as humans, and for which traits.

For evolutionary biologists, understanding the distribution of predictive versus constraints-induced plasticity in nature will help reveal the tradeoffs organisms make under resource-limited conditions, as well as the selective pressures that determine variation in tradeoffs across species. In addition, understanding the drivers of plasticity evolution is important for predicting how species will cope with environmental change [62], including through the evolution of plastic responses [33, 63, 64]. Accurately modeling responses to environmental change will require an understanding of the properties of the environment and the organism’s life history that determine whether and how different types of plasticity evolve.

### Genetics and genomics of developmental plasticity

#### Shared interests, current knowledge, and gaps in knowledge

**Figure 1.**
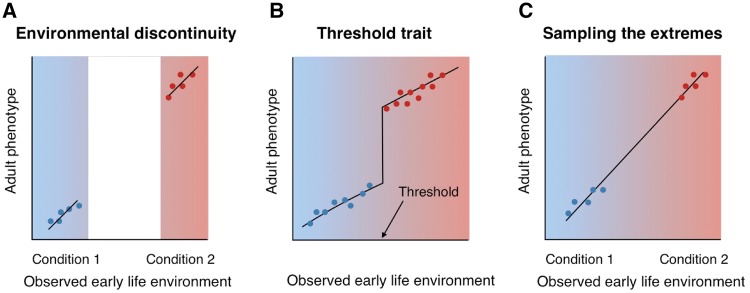
Polyphenisms (the appearance of discrete phenotypes in response to environmental variation) can arise in two ways: **(A)** when environmental variation is discontinuous so that only two regions of the reaction norm are ever expressed, or **(B)** when the organism exhibits a switch point or threshold value at which an alternate morph is produced. Modified from [112]; colored backgrounds indicate the nature of environmental variation, while dots indicate the environments in which organisms are sampled. Most research on the genes and molecular mechanisms underlying developmental plasticity has focused on organisms that naturally exhibit polyphenisms of the type depicted in (A) or (B), or has focused on the extremes of a phenotypic distribution that is naturally continuous, as depicted in **(C)**, such that an ‘artificial polyphenism’ is created for laboratory study. Few studies have examined a range of developmentally induced, continuous phenotypic variation of the sort typically exhibited by humans and other vertebrates, though this has been attempted in some cases (e.g. [87, 115, 169])

Identifying genes and genetic variants that contribute to plasticity is critical for understanding the evolutionary history of plastic traits, as well as sources of inter-individual variation in the capacity for plasticity. We focus on two questions related to this topic. First, what genes are involved in generating developmental plasticity within a given species? To date, work on this question has largely focused on polyphenisms, which occur when two or more discontinuous phenotypes are produced from the same genotype. Classic examples include caste differentiation in eusocial insects and seasonal morphs in butterflies, which have been well-studied at the molecular level [65–69]. A second, related question is: to what degree does genetic variation for plasticity itself exist among individuals of a given species? Work in both humans and non-human animals indicates that variation in the magnitude or direction of an environmental response is often a function of genotype, a phenomenon known as gene × environment (G × E) interactions [70–73] (Fig. 1).

**Figure 2.**
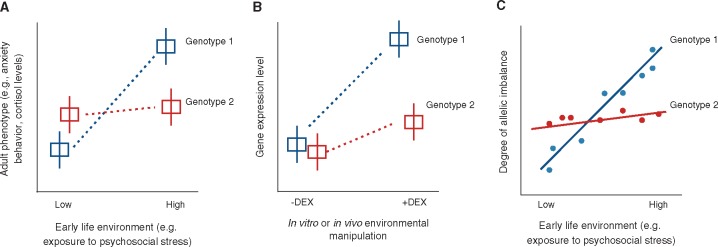
Approaches for mapping genetic variants that contribute to inter-individual differences in developmental plasticity. **(A)** If individuals of genotype 1 react differently to an early life environmental stressor than individuals of genotype 2, a gene by early environment (G × early E) interaction is implicated. Psychosocial stress has been a major focus of G × early E studies in molecular psychiatry, but the paradigm is generalizable to other early life environments. **(B)** Response eQTL studies can uncover G × early E interactions using *in vitro* or *in vivo* manipulations (such as treatment with dexamethasone, a synthetic glucocorticoid [170]). Under this paradigm, individuals with different genetic backgrounds, or cells cultured from these individuals, are experimentally exposed to two environments and the relationship between genotype and gene expression is assessed in both conditions. An interaction effect (as depicted here) would indicate that environmental sensitivity is genotype-dependent. **(C)** ASE measures the interaction between allelic imbalance (a difference in the expression levels of the two copies of a given gene within an individual) and the environment. If a particular early life environment alters the degree of allelic imbalance within heterozygotes for a marker variant (blue) but not homozygotes for the marker (red), this indicates that a G × early E interaction exists at a nearby regulatory genetic variant that is also heterozygous

In spite of these advances, major gaps in our knowledge remain. First, the genes involved in producing plasticity have only been identified in a few organisms (reviewed in [17, 69]), motivating expansion of this work to a larger set of species. Second, while many populations clearly harbor genetic variation for plasticity, few attempts have been made to identify the loci involved, especially using unbiased genome-wide approaches. For example, molecular psychiatrists have largely focused tests of gene × early environment interactions (referred to here as ‘G × early E’ interactions; Fig. 2A) on a handful of candidate genes [73, 74]. Such studies are often low powered and susceptible to unidentified confounds (e.g. population structure), leading to results that have been controversial and difficult to replicate [75]. Transitioning to well-powered whole genome scans, though challenging, would address these concerns [76], and provide a more nuanced view of the variants involved in a highly complex trait. Below, we discuss each of these gaps in knowledge, namely: (i) identifying genes that contribute to plasticity, and (ii) mapping individual-level differences in environmental sensitivity (i.e. G × early E interactions), with a focus on tractable molecular traits such as gene expression.

#### Identifying genes that contribute to developmental plasticity in diverse organisms

To understand the genetics of plasticity in a wide range of organisms—especially long-lived species that are not amenable to experimental manipulations—researchers will need to add additional approaches to their toolkit. One non experimental approach is to identify genetic differences between closely related species that exhibit differences in plasticity, specifically in cases where plasticity has been repeatedly gained or lost across a phylogenetic tree. For example, several *Daphnia* (water flea) species that develop helmets following early exposure to predators (a morphological change that protects these individuals from future predation) have sister species that do not exhibit this form of plasticity [58]. Similarly, closely related locust species vary in whether they display density-dependent polyphenisms [60], and aphid species differ in their capacity to produce winged versus unwinged morphs as a function of ecological conditions [77]. Scans for consistent genetic differentiation across multiple related species (or populations) that do versus do not display developmental plasticity could identify possible plasticity-related genes. Importantly, this study design has already proven useful for identifying the genetic basis of morphological and physiological traits in non-model systems [78, 79]. For example, Reid and colleagues compared genome sequences in 4 natural populations of killifish that had independently evolved tolerance to pollutants with four populations that had not, and found that genes involved in a single signaling pathway (the aryl hydrocarbonreceptor pathway) were repeated targets of selection in pollution-tolerant populations [78].

#### Mapping individual-level differences in environmental sensitivity on a genome-wide scale

Two genome-wide approaches could be harnessed to map G × early E interactions (Fig. 2B and C), both of which focus on how genetic variation affects gene expression, an environmentally sensitive molecular trait. The first approach is the study of ‘response eQTL:’ genetic variants that interact with the environment to influence gene expression levels (such that the magnitude or direction of the genotype-expression correlation varies across environments; Fig. 2C). Response eQTL have typically been identified *in vitro* by exposing cells to differing conditions and identifying variants that predict gene expression levels in a given condition (usually focusing on variants close to a given gene, known as ‘*cis*’ eQTL, to limit multiple hypothesis testing [80–82]).

Adapting the response eQTL framework to study developmental plasticity in other systems (including through *in vivo* approaches) is challenging but increasingly feasible as genomic resources accumulate for non-model systems. For example, one could identify genetic variants that affect gene expression in *Daphnia* clones exposed or unexposed to predators, or in spadefoot toad tadpoles fed a diet of shrimp versus detritus (an early life exposure that leads to striking differences in developmental trajectories) [83, 84]). Importantly, previous work in both humans and in non-model organisms has highlighted that early life experiences commonly affect long-term patterns of gene expression [17, 85–87], setting the stage for studies that investigate how these changes interact with genetic variation. In addition to designs that rely on *in vivo* exposures, culturing cells from non-model organisms is increasingly achievable. Thus, it may be possible to measure gene expression levels, in cells derived from the same individual, when these cells are cultured in the presence or absence of an *in vitro* cue that induces developmental plasticity in the system (e.g. hormone [68, 69] or kairomone [88, 89] cues).

A related approach for mapping G × early E interactions is the use of within-individual measures of allele specific gene expression (ASE). This approach capitalizes on the fact that when one allele of a gene within a heterozygous individual is overexpressed relative to the other allele of the same gene (i.e. when there is ‘allelic imbalance’), a nearby, *cis*-acting heterozygous regulatory variant must exist that modulates gene expression. If the degree of allelic imbalance within an individual varies across environments, a G × E interaction is implicated (Fig. 2B). ASE approaches have already been used to find variants associated with sensitivity to smoking, exercise, and medication use in human genome-wide gene expression datasets [90, 91], and early efforts have used ASE to identify genetic variation for developmental plasticity at candidate genes [92]. These approaches [91, 93–95] could be leveraged to investigate G × early E interactions in a range of organisms. Indeed, population-scale gene expression datasets are increasingly being collected for non-model organisms [96, 97], including in some long-term study populations [85, 98].

#### Gains from answering outstanding questions

Together, this work will clarify the genes involved in plasticity, as well as the loci that generate inter-individual variation. Doing so is important for understanding the biology of environmentally induced disease, and for identifying people who are particularly vulnerable to early life stressors. For example, recent work on another type of plasticity—the immune response—has leveraged the response eQTL framework to identify key transcription factors that mobilize the response to infection [82, 99], and to understand why individuals of European versus African genetic ancestry differ in their responses to pathogens [81]. Such approaches promise to improve our understanding of the mechanistic basis of plasticity, contributing to improved prediction and, over the long run, better options for therapeutic interventions.

For evolutionary biologists, the proposed work will identify genes involved in developmental plasticity in diverse species, and provide estimates of the prevalence, effect sizes, and genomic targets of G × early E effects. Such data will provide insight into the evolutionary history of plasticity, and will help reveal how early environmental perturbations alter the genotype–phenotype relationship. Further, understanding the genetic architecture of developmental plasticity (e.g. the number of genes that underlie this trait, as well as their effect sizes and pleiotropic or epistatic effects) will inform models of how rapidly plasticity can evolve when the environment shifts. Theoretical work in this area has assumed a simple architecture underlying plasticity (one or a few loci of large effect) [33]. If empirical data indicate this assumption is unwarranted [100, 101], we will need to radically readjust our models and predictions.

### The molecular mechanisms that mediate developmental plasticity

#### Shared interests, current knowledge and gaps in knowledge

What are the molecular mechanisms that allow early environmental variation to produce diverse phenotypes from static gene sequences? And further, following an early life exposure, which genes are targeted by such mechanisms (i.e. which genes are ‘differentially regulated’)? Answering these questions can advance our understanding of the causal chain linking environmental inputs with health or fitness-related outcomes.

Recent work on the molecular mechanisms that mediate developmental plasticity has focused most heavily on generating genome–wide gene expression or epigenetic data for individuals with known environmental histories. Here, we use the term ‘epigenetic’ to refer to a class of environmentally or developmentally sensitive mechanisms that can stably alter gene expression without changing the underlying DNA sequence (including DNA methylation, the best studied epigenetic mark to date [102–105]; histone modifications [102, 106, 107], chromatin accessibility; and non-coding RNAs [108]). Importantly, changes in the epigenome represent a major avenue through which environmental variation is translated into variation in organism-level traits. For example, groundbreaking work in laboratory mice found that maternal diet during pregnancy influences offspring methylation near the *agouti* gene, which in turn affects *agouti* gene expression, fur color, body mass, and susceptibility to diabetes [109, 110].

Studies like these have generated substantial interest in the degree to which changes in the epigenome explain early life effects on fitness-related traits [111–114]. However, several key gaps remain. First, as with studies of the genes involved in plasticity, studies of early environmental effects on genome-wide gene regulation (i.e. epigenetic marks or gene expression levels) have been concentrated in a handful of systems and contexts: model organisms and humans that experienced extreme early life challenges (e.g. famine, institutionalization, or child abuse) [17, 69, 104, 105, 115–117]). As a result, general principles governing plastic responses at the molecular level—including how they evolve and function in nature—remain unclear. Second, a robust body of work has shown that environmentally responsive gene regulatory mechanisms are sensitive to stimuli across the life course, not just in early life [118–120]. Thus, a key outstanding question is: to what degree are epigenetic marks and gene expression patterns stably affected by conditions in early life (e.g. during the prenatal, neonatal, and juvenile periods) versus reversible in adulthood? Below we discuss approaches for addressing these missing pieces.

#### Early life environmental effects on gene regulation in diverse species

Molecular studies of captive or laboratory animals can provide powerful insight into causality, but two common features of their design can limit their generality. First, animal models of early adversity are often extreme, and thus differ from the conditions animals have encountered over their evolutionary history [87, 121–124]. Second, lab-based studies frequently compare the outliers of an early environmental distribution in a case/control framework, an approach that fails to address common variation that lies near the mean (Fig. 1). Work in human medical epigenetics has followed a similar pattern, and has largely focused on early life challenges with major health consequences (e.g. famine, child abuse or institutionalization [105, 115, 117, 125, 126]). Currently, the degree to which responses to ‘atypical’ stressors (e.g. evolutionarily novel, rare, or extreme stressors) recapitulate responses to stimuli that organisms have encountered repeatedly throughout their evolutionary history is unknown. Importantly, theory suggests that animals evolve plasticity in stress response pathways over many generations of stabilizing selection, and extreme or novel environments may break down or ‘decanalize’ such responses, increasing variability in unexpected ways [127–129]. Going forward, we therefore suggest dual emphases on both (i) understanding gene regulatory responses to atypical early environmental perturbations with large health or fitness effects, and (ii) covering the spectrum of naturally occurring early environmental variation. This dual approach will allow us to understand whether regulatory responses to naturally occurring stressors are conserved, and how such systems are perturbed when the stressor falls outside the species’ evolutionary range.

Developing a general picture of the gene regulatory consequences of early environmental stress would be greatly accelerated by transcriptomic and epigenomic datasets from diverse, longitudinally studied populations. Studies of humans and non-human mammals are likely to be mutually informative, as epigenetic mechanisms are largely conserved across these species [130, 131]. This strategy has already been modeled by a handful of studies focusing on gene expression or DNA methylation [85, 98, 132, 133], providing important proof of principle. Work on other epigenetic marks such as histone modifications, chromatin accessibility and non-coding RNAs is less advanced, presumably because these marks are more challenging to measure outside of the laboratory (e.g., for certain marks, protocols must be performed on large numbers of live cells immediately upon collection). However, genomic protocols are rapidly becoming more streamlined and optimized for low-input [134–136], making it increasingly possible to study multiple epigenetic marks in longitudinally followed populations of wild animals or remotely living humans.

#### The timing and stability of environmental epigenetic effects

Initial work on epigenetic marks, especially DNA methylation, emphasized the stable effects of events that occurred during specific early life critical periods, a phenomenon referred to as ‘embedding’ or ‘programming’ [103, 137–139]. However, recent work has shown that conditions throughout early life (i.e. the period between conception and reproductive maturity [8]) and in adulthood can both have potent effects on the epigenome [118, 120, 140]. This duality implies that it may be possible to reverse (or further exacerbate) epigenetic changes induced by early adversity, depending upon the environment later in life (as in [141]). However, the degree to which variation in any epigenetic mark is determined by early life versus adult conditions remains poorly understood. Early studies that have tackled this important question (all focused on DNA methylation in humans) have suffered from low power, confounds between adult environments and health habits, and a reliance on retrospective self-reporting [115, 116, 138].

Here again, long-term studies of natural or captive animal populations—where blood or other tissue is routinely collected from individuals followed over the life course—could be leveraged to circumvent problems posed by human population studies. In addition, such work could be fruitfully complemented by (i) prospective, longitudinal studies of human cohorts with repeated biological sampling (many of which are in progress, and will be invaluable once completed [142]); and (ii) experiments in lab-based systems. For example, studies of laboratory rodents and captive rhesus macaques have identified epigenetic changes following early life manipulations [102, 103, 143, 144], but they have rarely been extended to coupled manipulations of both early life and adult environments (but see [145]). Additionally, studies of birds that produce altricial hatchlings offer the ability to experimentally investigate early life stressors that are ecologically relevant, and to combine these with the ability to monitor longitudinal fitness outcomes and collect molecular samples [146–150]. In systems such as these, and others that share similar features, researchers could expand on study designs used to investigate evolutionary explanations for early life effects [16], where individuals are exposed to high- or low-quality early environments followed by high- or low-quality adult environments in a ‘fully factorial’ design. In this case, however, the phenotype of interest would be genome-wide epigenetic patterns, rather than fitness.

#### Gains from answering outstanding questions

For human health researchers, understanding the gene regulatory effects of early life stressors—both typical and atypical—is essential for developing generalizable, predictive frameworks for environmentally induced disease. Epigenetic studies of wild animals have an important potential role here: they can illuminate whether organisms living in their natural environments respond to stressors in ways that recapitulate responses in laboratory animal models or humans. Further, such comparisons can help to empirically test whether certain modern early life exposures, which may be atypical relative to human evolutionary history, perturb evolutionarily conserved gene regulatory programs in ways that lead to poor health outcomes. With respect to timing, understanding the genome-wide stability of early environment-induced epigenetic changes will reveal the degree to which early adversity can be exacerbated or reversed by adult conditions. This information is key for understanding when interventions would be most meaningful.

Answers to these same questions are also important for testing and advancing evolutionary theory. As noted earlier, some types of plasticity (e.g. predictive plasticity, Box 1) are expected to evolve only under particular environmental scenarios. For example, when the environment varies on a timescale greater than the lifespan of the organism and not predictable during development, a risk-spreading strategy known as ‘diversifying bet-hedging’ is expected to evolve [33, 151, 152]. Under this strategy, individuals randomly develop one of several phenotypes, each of which is beneficial in one possible adult environment. Conditions selecting for both predictive plasticity and bet-hedging are common in nature [33, 153] and thus an empirical study of whether epigenetic mechanisms adhere to these predictions should be feasible in natural systems. Specifically, if the environment selects for irreversible, predictive responses to early conditions, do we see a strong and stable correlation between early environmental variation and epigenetic variation? And where bet-hedging is the favored form of plasticity, are epigenetic profiles randomly allocated among individuals at birth in a stable manner? In this way, epigenomic data could be used to test and refine theories of the evolution of developmental plasticity, and to understand whether molecular mechanisms follow theoretical predictions about plasticity in organism-level traits.

## CONCLUSIONS

We have highlighted key outstanding questions in developmental plasticity research, along with suggestions for how to answer them. Our list is by no means exhaustive, but it is meant to ignite conversations between evolutionary and health researchers, and to highlight the acute relevance of work in each field to that in the other. The gains of establishing cross-disciplinary ground in developmental plasticity research will only be realized when human health researchers and evolutionary biologists go beyond the simple recognition that they share overlapping interests, and begin to critically evaluate their own work in the light of research in the other tradition.

The emergence of cross-disciplinary journals such as *Evolution, Medicine, and Public Health* represents a critical step in the right direction. However, researchers must also seek out papers and conversations with colleagues from unfamiliar disciplines. For instance, evolutionary researchers studying wild animals must look to the vast human literature on early life effects to inform their work, as several groups have already done in compelling ways [6, 15, 27, 149, 154, 155]. The power of merging evolutionary frameworks with questions and literature related to human health is also well demonstrated by research focused on longitudinal studies of human populations, where groundbreaking work has begun on evolutionary explanations for developmental plasticity and the developmental origins of disease [14, 28, 29, 156, 157]. At the same time, human health researchers must appreciate the insight provided by work in non-model animal systems, exemplified by several recent studies [158, 159]. More ambitiously, by collaborating on theoretical and empirical work (as in [9, 160–162]), human health and evolutionary researchers can increasingly leverage their disciplinary expertise to push forward the boundaries of our knowledge.
